# Supplementary material for: Multi‐dataset identification of innovative feature genes and molecular mechanisms in keratoconus
Source: J Cell Mol Med. 2024 Sep 19;28(18):e70079. doi: 10.1111/jcmm.70079 (PMC11412914; doi:10.1111/jcmm.70079)
Supplement: Supplementary file 2 — Material S2. [file JCMM-28-e70079-s002.zip › 7.GSEA analysis/5.RNF217-AS1/KEGG_VASOPRESSIN_REGULATED_WATER_REABSORPTION.html]

Details for gene set KEGG\_VASOPRESSIN\_REGULATED\_WATER\_REABSORPTION[GSEA]

|  || Dataset | RNF217-AS1.group.cls#h\_versus\_l |
| Phenotype | group.cls#h\_versus\_l |
| Upregulated in class | l |
| GeneSet | KEGG\_VASOPRESSIN\_REGULATED\_WATER\_REABSORPTION |
| Enrichment Score (ES) | -0.38544816 |
| Normalized Enrichment Score (NES) | -1.3668531 |
| Nominal p-value | 0.108829565 |
| FDR q-value | 0.35221142 |
| FWER p-Value | 0.899 |
Table: GSEA Results Summary

  

Fig 1: Enrichment plot: KEGG\_VASOPRESSIN\_REGULATED\_WATER\_REABSORPTION      
 Profile of the Running ES Score & Positions of GeneSet Members on the Rank Ordered List

  

| SYMBOL | TITLE | RANK IN GENE LIST | RANK METRIC SCORE | RUNNING ES | CORE ENRICHMENT || 1 | AQP3 | na | 7504 | 0.140 | -0.1773 | No |
| 2 | DCTN5 | na | 7990 | 0.128 | -0.1557 | No |
| 3 | DYNC1LI2 | na | 9427 | 0.097 | -0.1701 | No |
| 4 | RAB5C | na | 11541 | 0.062 | -0.2138 | No |
| 5 | CREB3 | na | 11909 | 0.057 | -0.2085 | No |
| 6 | NSF | na | 13636 | 0.035 | -0.2487 | No |
| 7 | DYNLL1 | na | 14716 | 0.022 | -0.2736 | No |
| 8 | RAB11B | na | 14785 | 0.021 | -0.2696 | No |
| 9 | VAMP2 | na | 14994 | 0.019 | -0.2702 | No |
| 10 | STX4 | na | 15117 | 0.018 | -0.2688 | No |
| 11 | DYNC1H1 | na | 15310 | 0.016 | -0.2699 | No |
| 12 | DYNC1I2 | na | 16121 | 0.008 | -0.2911 | No |
| 13 | CREB1 | na | 16423 | 0.004 | -0.2985 | No |
| 14 | CREB3L3 | na | 16617 | 0.003 | -0.3034 | No |
| 15 | ADCY9 | na | 17206 | -0.004 | -0.3193 | No |
| 16 | DCTN6 | na | 17745 | -0.008 | -0.3325 | No |
| 17 | GNAS | na | 17807 | -0.009 | -0.3317 | No |
| 18 | ADCY6 | na | 19355 | -0.024 | -0.3697 | No |
| 19 | DCTN2 | na | 19382 | -0.024 | -0.3638 | No |
| 20 | CREB3L2 | na | 20073 | -0.031 | -0.3751 | No |
| 21 | RAB5A | na | 20434 | -0.034 | -0.3759 | Yes |
| 22 | DYNLL2 | na | 20501 | -0.035 | -0.3680 | Yes |
| 23 | CREB3L1 | na | 20661 | -0.036 | -0.3624 | Yes |
| 24 | DCTN4 | na | 21204 | -0.042 | -0.3663 | Yes |
| 25 | CREB3L4 | na | 21836 | -0.049 | -0.3709 | Yes |
| 26 | DCTN1 | na | 21980 | -0.051 | -0.3609 | Yes |
| 27 | ARHGDIA | na | 22706 | -0.058 | -0.3655 | Yes |
| 28 | ADCY3 | na | 22794 | -0.059 | -0.3515 | Yes |
| 29 | AVP | na | 22886 | -0.060 | -0.3372 | Yes |
| 30 | PRKACA | na | 23066 | -0.063 | -0.3249 | Yes |
| 31 | RAB5B | na | 23086 | -0.063 | -0.3079 | Yes |
| 32 | ARHGDIB | na | 23477 | -0.068 | -0.3003 | Yes |
| 33 | AVPR2 | na | 23857 | -0.073 | -0.2908 | Yes |
| 34 | RAB11A | na | 23955 | -0.074 | -0.2729 | Yes |
| 35 | AQP2 | na | 26185 | -0.110 | -0.3064 | Yes |
| 36 | PRKACB | na | 26457 | -0.116 | -0.2820 | Yes |
| 37 | DYNC1LI1 | na | 27236 | -0.131 | -0.2680 | Yes |
| 38 | DYNC2H1 | na | 27914 | -0.146 | -0.2467 | Yes |
| 39 | DYNC1I1 | na | 28513 | -0.162 | -0.2186 | Yes |
| 40 | DYNC2LI1 | na | 28687 | -0.168 | -0.1769 | Yes |
| 41 | PRKX | na | 31362 | -0.257 | -0.1823 | Yes |
| 42 | AQP4 | na | 32194 | -0.302 | -0.1220 | Yes |
| 43 | CREB5 | na | 32518 | -0.325 | -0.0406 | Yes |
| 44 | PRKACG | na | 33174 | -0.374 | 0.0448 | Yes |
Table: GSEA details [plain text format]

  

Fig 2: KEGG\_VASOPRESSIN\_REGULATED\_WATER\_REABSORPTION      
 Blue-Pink O' Gram in the Space of the Analyzed GeneSet

  

Fig 3: KEGG\_VASOPRESSIN\_REGULATED\_WATER\_REABSORPTION: Random ES distribution      
 Gene set null distribution of ES for **KEGG\_VASOPRESSIN\_REGULATED\_WATER\_REABSORPTION**
